# “What Made My Eating Disorder Worse?” The Impact of the COVID-19 Pandemic from the Perspective of Adolescents with Anorexia Nervosa

**DOI:** 10.3390/nu15051242

**Published:** 2023-03-01

**Authors:** Susanne Gilsbach, Beate Herpertz-Dahlmann

**Affiliations:** Department of Child and Adolescent Psychiatry, Psychosomatics and Psychotherapy of the RWTH Aachen, Neuenhofer Weg 21, 52074 Aachen, Germany

**Keywords:** COVID-19, adolescents, anorexia nervosa, eating disorder, remote treatment

## Abstract

(1) Background: the COVID-19 pandemic and subsequent confinements have led to a dramatic increase in anorexia nervosa (AN) in adolescent patients, whereas the effect on symptom severity and the influencing factors are not yet clear, especially not from the adolescents’ perspective. (2) Methods: from February to October 2021, 38 adolescent patients with AN completed an adjusted version of the COVID Isolation Eating Scale (CIES), a self-report questionnaire asking for ED symptomatology before and during the COVID-19 pandemic and for their experiences with remote treatment. (3) Results: patients reported a significant negative impact of confinement on ED symptoms, depression, anxiety, and emotional regulation. During the pandemic, engagement with weight and body image was related to social media, and mirror checking increased. The patients were more preoccupied with cooking recipes and had more eating-related conflicts with their parents. However, the differences in the amount of engagement with social media actively glorifying AN before and during the pandemic did not remain significant after correction for multiple comparisons. The minority of patients who received remote treatment found it to be only limitedly helpful. (4) Conclusions: from the patients’ perspective, the COVID-19 pandemic-associated confinement had a detrimental effect on the symptoms of adolescent patients with AN.

## 1. Introduction

The COVID-19 pandemic had a detrimental effect on the mental health of children and adolescents (see for example [1,2]). In addition to an increase in more general mental health problems, such as anxiety and depression [2], the prevalence of eating disorders (ED), especially anorexia nervosa (AN), has increased all over the Western world and across all age groups during the COVID-19 pandemic (see [3] for an overview). Since the peak onset of AN occurs in adolescence [4], the pandemic-associated impact on the number and symptom severity of adolescent patients with AN is of particular interest.

Gilsbach et al. [5] found a pandemic-associated increase in admissions of adolescents with AN to specialized treatment centers across Europe. Furthermore, the specialists for AN perceived their patients as being affected negatively by the COVID-19 pandemic on a variety of symptoms, among others concern about weight, diet and body image, loneliness, agitation, nervousness, and frequency of physical activities. Similarly, Herpertz-Dahlmann et al. [6] described a 40% increase in hospitalizations because of childhood AN and a more than 30% increase because of adolescent AN in a nationwide sample in Germany. The high admission rates were not, however, caused by an increase in readmission rates, but were more likely due to COVID-19 induced new onsets of AN or more severe courses of the disorder with more patients needing hospital admissions. Interestingly, there was also an increase in childhood male AN patients in this sample. Otto et al. [7] reported twice as many medical admissions to a pediatric hospital in the US due to EDs during the COVID-19 pandemic compared to the corresponding pre-pandemic time frame. Accordingly, Agostino and Burstein [8] found stable pre-pandemic numbers of AN cases in a Canadian pediatric tertiary care hospital and a steep upward trend with more diagnosed cases and more hospitalizations for newly diagnosed patients during the first wave of the pandemic.

However, when examining AN symptom severity and influencing factors associated with the COVID-19 pandemic, research results have been contradictory (see [9] for an overview). Most researchers have found a pandemic-related worsening of adolescent AN symptoms using medical parameters such as the body mass index (BMI) and heart rate at admission [8,10], as well as by assessing psychopathology (see, for example [11]).

Others have found an increase in AN cases but no changes in symptom severity with regard to medical parameters such as incidences of bradycardia, postural hypotension, requirements for electrolyte supplementation, nasogastral feeding, BMI, and amenorrhea [12,13]. Describing psychopathology, Akgül et al. [14] found that 42.1% of their adolescent sample (mostly patients with AN, some with bulimia nervosa) even reported a positive effect of the lockdown on their ED symptoms, and only 21.1% reported worsening. Accordingly, in an adult sample consisting of all types of EDs, only ¼ of the patients reported a worsening of symptoms during the lockdown [15].

Furthermore, research on the exact mechanisms of the COVID-19 pandemic on ED symptoms has been scarce and mostly qualitative. In a survey with 159 former patients with AN (mean age 22.4 years old, age range 14–62), approximately 70% reported that eating, shape, and weight concerns, a drive for physical activity as well as loneliness, sadness, and inner restlessness all increased during the pandemic. Access to in-person psychotherapy and visits to general practitioners (including weight checks) decreased by 37% and 46%, respectively [16]. Branley-Bell and Talbot [17] revealed in a qualitative study of patients with all types of EDs and across all ages several key themes affecting ED symptoms negatively, such as the “disruption to living situation, increased social isolation and reduced access to usual support networks, changes to physical activity rates, reduced access to healthcare services, disruption to routine and perceived control, changes to relationship with food, increased exposure to triggering messages”, while positively mentioned was an increase in digitally available social support systems. Correspondingly, in interviews with adolescent patients suffering from AN discussing experiences with COVID-19 confinement, the emerging themes consisted of “restrictions of personal freedom, interruption of the treatment routine, changes in ED und other psychopathology”. “Less stress, more family time, autonomy and self-organization skills” emerged as opportunities arising from the pandemic [18].

In sum, the results regarding pandemic-related effects on AN symptomatology have been contradictory, and quantitative data regarding mediating factors have been scarce.

In the current study, we aimed to assess changes in ED symptom severity due to the COVID-19 pandemic and to determine the contributing factors in an adolescent sample. We chose the only currently existing validated self-report questionnaire to assess the impact of confinement on EDs, the COVID Isolation Eating Scale (CIES) [19], and we adjusted it for adolescent patients. We hypothesized that self-reported symptom severity would be worse overall during the confinement than before the pandemic. More precisely, we hypothesized that there would be a pandemic-related negative impact on different features contributing to the severity of AN, such as eating behaviors, emotional regulation, and an increase in depressive and anxious symptomatology. We also anticipated an increase in body-related media consumption, as well as eating-related conflicts with parents. Finally, with a view toward future treatment recommendations, we assessed how many of our patients had received remote treatment and how effective they had found it to be.

## 2. Materials and Methods

### 2.1. Participants

From February to October 2021, all patients (*n* = 40) who were treated in the Department for Child and Adolescent Psychiatry, Psychosomatics, and Psychotherapy of the RWTH-Aachen, Germany for an eating disorder (ED) (anorexia nervosa (AN) or bulimia nervosa (BN) according to DSM-5) completed an adjusted version of the CIES [19] as part of the diagnostic routine. The investigation period began during the second lockdown, which started with minor restrictions in November 2020 and included major restrictions including school closures from January 2021 until May 2021. The preceding first lockdown including school closures dated from March 2020 to May 2020.

### 2.2. Original Questionnaire and Adjustments

The CIES is a self-report questionnaire to assess the impact of confinement on the psychopathology of patients with an ED during the COVID-19 pandemic. It is the only validated questionnaire of this type. The CIES asks for sociodemographic information, as well as current height and weight and weight before the onset of the COVID-19 pandemic, and it is then subdivided into four sections. The first section consists of items about the “circumstances during confinement” with questions about the living conditions, work, the financial situation and whether the patient was ill with COVID-19 or knew someone who was (8 items). The second section contains questions regarding the current diagnosis, comorbidities, and items assessing the “effects of confinement on eating disorder symptoms” (10 items; concerns about weight, attempts to reduce the quantity of eating and the number of meals, bingeing/purging, use of laxatives/diuretics, and exercise or other activities to control weight). The third section assesses “reactions to confinement” (34 items, e.g., emotional eating, anxiety, depression, dysfunctional thoughts, and addictive behaviors). The 10 items of section two and all items of section three are answered on a 5-point Likert scale (never–always) and should be answered twice, respectively, “before confinement” and “currently”. The fourth section contains an evaluation of experiences with remote therapeutic interventions, asking about feasibility, acceptance, and satisfaction on a five-point Likert scale (totally disagree–totally agree) (10 items) and open questions about challenges, strengths, and weaknesses of remote treatment (3 items).

According to [19], the CIES measures five underlying factors: factor 1 (F1)—**eating disorder-related symptoms**, represented by the 10 items of section two; factor 2 (F2)—effects of confinement on the **eating-related style** (10 items from section three, such as eating for comfort, craving for food, feeling ashamed for eating); factor 3 (F3)—**anxiety and depressive symptoms** (11 items from section three, such as sleep problems, upsetting thoughts, loneliness, limited social contact, health concerns related to COVID-19, or sexual problems); factor 4 (F4)—**emotional regulation** (5 items from section three, such as irritability, aggression against self or others, and feeling of loss of control); and factor (F5), which is defined by the 10-item **evaluation of remote treatment**. Please refer to [19] for further information about the CIES, including its psychometric properties, which were good to excellent (Cronbach’s alpha between 0.81 and 0.92) for the Spanish version. The CIES was translated into 19 languages.

Since the original CIES was developed for adults with a variety of eating disorders, we made slight adjustments to the questionnaire for our purposes. We omitted questions concerning obesity and its consequences, such as diabetes mellitus, since these aspects were not relevant for our patient group. Furthermore, we adjusted the demographic questions according to the age and life situations of our patients, e.g., we asked about school and parents, not about work and partners. Finally, we added questions regarding social media use and conflicts with parents about eating behaviors. Our adjustments, however, did not prevent the calculation of the main factors since all relevant items remained in the original version and we analyzed the added questions separately.

### 2.3. Statistical Analyses

For all statistical analyses, we used IBM SPSS Statistics software, version 27.0 for Windows (Released 2020; IBM Corp., Armonk, NY, USA).

For comparisons of pre-confinement with post-confinement values, we used the paired t-test. Furthermore, we computed estimations of effect sizes using Cohen’s d coefficient (│d│ < 0.2 no, │d│ > 0,2 low, │d│ > 0.5 medium, │d│ > 0.8 high effect).

F5 was not computed due to the lack of a comparison group, but the mean scores for the items belonging to F5 were depicted separately.

## 3. Results

### 3.1. Sample Characteristics

Thirty-eight patients suffered from AN, and two suffered from BN. The two patients with BN were excluded from the analysis due to the small number. Please see Table 1 for sociodemographic and clinical information.

All patients lived with their families at the time of confinement. Fourteen (36.8%) underwent homeschooling, twenty-two (57.9%) received a combination of homeschooling and in-person schooling, one (2.6%) went to school in person, and one answer was missing (2.6%). At the time of completion of the questionnaire, none of the patients had suffered from COVID-19, and four (10.5%) had family members or friends who had experienced COVID-19. One (2.6%) patient reported financial problems due to the COVID-19 pandemic.

### 3.2. Changes in AN Symptomatology

The patients’ current mean BMI was significantly lower than that before the onset of confinement. There was also a significant increase in scores from pre-measures to current measures, indicating an increase in the symptom burden for all ED domains, except that represented by F2, “changes in eating style” (Table 2).

### 3.3. Changes in Social Media Use, Mirror Checking, Cooking, and Conflicts with Parents

There was a significant increase in the amount of overall social media use. However, the difference in the amount of engagement with social media actively glorifying AN before and during the pandemic did not remain significant after correction for multiple comparisons. Patients reported an increase in mirror checking, engaging with cooking recipes, and conflicts with their parents due to eating. The frequency of cooking, as well as conflicts with parents not due to eating, remained unchanged (Table 3).

### 3.4. Remote Treatment

**Figure 1:**
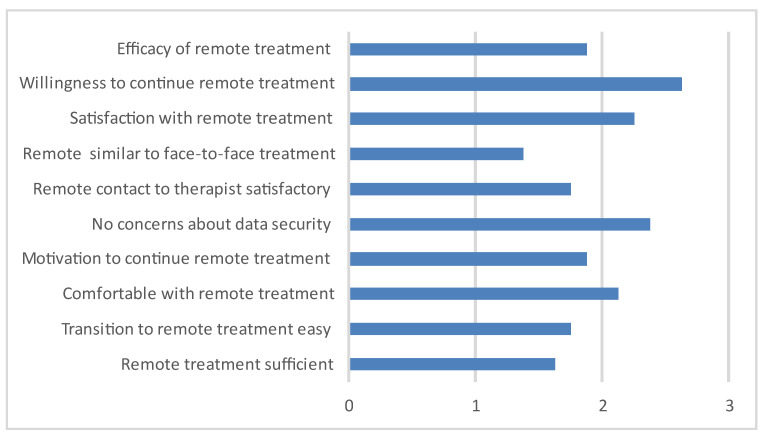
Evaluation of remote treatment (*n* = 8, 0 = totally disagree, 4 = totally agree).

Eight out of thirty-eight patients received remote treatment during the pandemic. The evaluation is depicted in Figure 1.

As challenging aspects of the remote treatment, the participants mentioned a lack of privacy at home, digital obstacles, the missing division between everyday life and the therapeutic setting, and greater personal distance, leading to less open interaction and more opportunities to dissimulate weight loss issues or other problems. As advantages, the opportunity to continue treatment during lockdown and the lack of a need to drive to the treatment setting were mentioned.

## 4. Discussion

This study is the only study that examined changes in AN symptomatology in adolescent patients during the COVID-19 pandemic using a validated questionnaire, asking for direct pre-/post-comparisons and focusing on the adolescents’ perspective.

Overall, we found a detrimental impact of COVID-19 pandemic-associated changes on the psychopathology of adolescent patients with AN.

As hypothesized, we found a significant increase in ED-related symptoms. This finding is supported by most of the comparable studies (e.g., [9]), but contrasts with the results of Fernandez-Aranda et al. [19], who developed the CIES in an adult sample and found a decrease in ED symptomatology. This discrepancy might be due to a difference in the participants’ ages or to the temporal gap of one year between the studies, meaning the investigation periods during the first in contrast to the second lockdown, respectively. Younger patients were shown to be especially prone to developing AN during the COVID-19 pandemic, hinting at the greater vulnerability for this age group [5,6].

There was no difference in eating-related style, which is not surprising since the items belonging to this factor measure bingeing/grazing/craving behaviors, and all included patients wo suffered from the restrictive subtype of AN; therefore, binging/craving/grazing are usually not one of their main concerns.

The significant, negative impact of the pandemic on feelings of anxiousness and depression reported by our patients mirrors well the emotional burden caused by confinement, not only for patients with AN [11,16] but also for youths in general [1,2,20]. In a German nationwide longitudinal survey, over two thirds of the participating children and adolescents between 11 and 17 years reported being burdened by the pandemic and suffering from a significantly lower health related quality of life [1]. Accordingly, we found an increase in emotional dysregulation, including irritability, which has also been described previously by Tombeau Cost et al. [21] in children and adolescents with and without mental health problems during the pandemic. However, depressive and anxiety disorders are also frequent comorbid disorders in AN; thus, a differentiation between the effects of the pandemic and AN-associated comorbid symptomatology is difficult, although the patients reported a pandemic-induced increase.

The digital media consumption of patients with AN, especially associated with body weight and shape, increased distinctly between the pre-pandemic and peri-pandemic times. This finding was not unexpected since more spare time and fewer activities might lead to a higher engagement in screen time [22,23]. Among the major physical health concerns in the Western world related to the COVID-19 pandemic-associated lockdowns were weight gain and a subsequent increase in obesity, for both children and adolescents [24,25]. Accordingly, the number of social media platforms with dietary and workout recommendations increased and gained broader interest, and warnings regarding “quarantine-15”, referring to a typical weight gain of 15 pounds due to the quarantine, could be found on several internet and social media pages [26]. Thus, children and adolescents susceptible to AN not only had more time to indulge in screen time but were also at a substantially increased risk of being confronted with the topic of weight loss in the digital world. The etiological contribution of AN-glorifying websites (“Pro-ANA”) to the development of AN, especially in female teenagers, has already been established [27]. In addition to the digitally fueled fears of weight gain due to a lack of physical activity, we suspect the changes in social media consumption to have been an important mediating factor in the development and deterioration of AN in children and adolescents during the pandemic.

Furthermore, our participants reported more mirror checking, more engaging with recipes and more eating-related conflicts with their parents. This outcome is likely due to them spending more spare time at home. There was no relevant increase in conflicts other than eating-related conflicts. These findings support some of the pathways proposed by Rodgers et al. [28] through which the COVID-19 pandemic might increase ED risks and symptoms, the pathways namely being social media consumption, negative affect, disruption to daily activities, and social isolation. The disruption of daily routines in combination with the social isolation resulting from confinement is thought to lead to a decrease in resilience and adaptive coping strategies, and an increase in time spent with possibly harmful social media consumption and worrying thoughts regarding body image and weight. Furthermore, fears of contagion might lead to changes in diet with the aim of boosting immunity, simultaneously and involuntarily increasing the risk of developing an ED. Finally, stress and negative affect in general might contribute to the increase in risk for an ED [28].

There was a significant difference in self-reported BMI before and during confinement, with the BMI “before” being within the normal range and that “during” indicating being underweight. In principle, BMI could be interpreted as a medical marker of the disease severity of AN [8,10].

Although the pandemic had already lasted for one year at the time of our study, only approximately one-fifth of the participants had received remote treatment. Furthermore, satisfaction with digital treatment was mediocre, and was not regarded as a good substitute for in-person care, neither was it seen as a fit substitute. This result corresponds to [17] who found that patients with AN were the least comfortable with remote treatment, when compared to patient groups with other EDs, such as bulimia nervosa or obesity. [18] also confirmed that adolescent patients with AN preferred face-to-face appointments, also with regard to being able to leave difficult subjects “in the doctor’s office”. When deprived of possibility of in person therapy, patients chose a video-connection over an audio-only connection, because they regarded facial expressions and gestures as important for the therapeutical relationship. However, the overall willingness to continue receiving remote treatment when there was not alternative was good. 

This study has several limitations. The CIES was originally developed and validated for adults in a Spanish sample and was supposed to distinguish between participants with different ED diagnoses, such as AN, bulimia nervosa, and obesity. Our sample only comprised adolescents with restrictive AN. Moreover, the questionnaire was not validated in a German sample. However, it had been used in an international sample with 829 participants from 11 countries including 146 German-speaking patients and differentiated well between pre- and post-COVID eating disorder and non-eating disorder symptoms [29]. Furthermore, the participants’ assessments of the time before the COVID-19 pandemic were made post hoc by memory recall at the same time as the assessment of the current situation. In addition, the comorbid diagnoses were only reported by the patients themselves, not assessed by experts. However, when we compared the self-reported diagnoses of those participants, who had also been treated as inpatients, to those of the clinicians, there was no significant difference. Finally, the sample size was rather small, and only a minority of the participants received video treatment.

## 5. Conclusions

In summary, we found a deterioration of AN symptomatology and general psychopathology during the COVID-19 pandemic. Mediating factors seemed to include the general psychological burden caused by pandemic-associated restrictions, in addition to fears of weight gain, increased exposure to media glorifying a low body weight, mirror checking, and the medial topic of healthy and low carb foods. Although remote treatment on the basis of our results cannot be considered equivalent to in-person care, the broadening of digital treatment offers in times of confinement remains an important means of care for patients with AN. However, further research on its effectiveness is still needed.

## Figures and Tables

**Table 1:** Demographic and clinical characteristics of 38 adolescent patients with AN. All information are self-reports taken from the questionnaire.

Mean Age	Years (SD) 15.1 (1.7), Range 12–18	
Type of treatment	Current (*n*/%)	Before the onset of the COVID-19 pandemic (*n*/%)
None	0	29/76.3
Outpatient	9/23.7	7/18.4
Inpatient	27/71.1	1/2.6
Day patient	1/2.6	0
Home treatment	1/2.6	0
Not specified	0	1/2.6
Average length of treatment in months (SD)	-	11.6 (16.3) range: 2–48
Comorbidities (self-report)		
Depressive symptoms	12/31.6	
Anxiety disorder	5/13.2	
Social phobia	5/13.2	
Obsessive–compulsive disorder	2/5.3	
Other	3/7.9	
More than one comorbidity	10/26.3	

**Table 2:** Comparison of differences in BMI and CIES factor scores (pre-pandemic versus current).

	Pre		Current			
*n* = 38	Mean	(SD)	Mean	(SD)	*p*	│d│
BMI	20.1	(3.1)	17.4	(2.3)	**<0.001**	0.78
Impact on ED symptoms (F1)	11.8	(5.4)	18.2	(5.2)	**<0.001**	0.99
Changes—eating related style (F2)	7.5	(5.8)	8	(6.1)	0.65	0.08
Changes—depression/anxiety (F3)	11.6	(7.7)	18.4	(8.7)	**<0.001**	0.91
Changes—emotional regulation (F4)	5.8	(3.7)	8.5	(5.0)	**<0.001**	0.98

Paired *t*-tests for “pre” versus “current” for BMI and the five underlying factors “impact on ED symptoms” (F1), “eating related style” (F2), “depression/anxiety” (F3), and “emotional regulation” (F4). After correction for multiple testing, *p*-values < 0.01 remain significant and are printed in bold.

**Table 3:** Added questions to regarding behaviors influencing symptomatology.

	Pre		Current			
	Mean	(SD)	Mean	(SD)	*p*	│d│
Engaging in social media glorifying AN (“pro-ANA”)	0.3	1.0	0.6	1.1	0.016	0.41
Following models and influencers on social media	1.9	1.2	2.4	0.9	**0.002**	0.54
Using apps for weight loss	0.8	1.2	1.8	1.6	**0.002**	0.56
Preoccupied with cooking recipes	2.0	1.2	2.8	0.9	**0.001**	0.62
Preoccupied with cooking	2.3	0.8	2.6	1.2	0.201	0.21
Mirror checking	2.5	0.8	3.2	1.1	**<0.001**	0.65
Conflicts with parents—due to eating	1.3	1.2	2.6	1.0	**<0.001**	0.95
Conflicts with parents—not due to eating	1.7	0.7	1.7	1.0	0.474	0.12

Answers were given on a 4-point Likert scale (0 = never–4 = always). Each item was answered twice: retrospectively for the situation before the pandemic (“pre”) and for the current situation at the time of completion of the questionnaire (“current”). After correction for multiple testing, the *p*-values < 0.006 remain significant and are printed in bold.

## Data Availability

The data that support the findings of this study are available on request from the corresponding author, [BHD].
